# Cystic Echinococcosis in Agro-Pastoral Regions: A 10-Year Retrospective Study (2015–2024) and the Case for a One Health Approach

**DOI:** 10.3390/tropicalmed11070180

**Published:** 2026-06-28

**Authors:** Messaoud Bouragba, Samir Abdellaoui, Sarah Saci, Nasir A. Ibrahim, Mohammed Saad Aleissa, Nosiba S. Basher, Nesrine Goumich, Sundes Rabia Khelifa, Meriem Aissou, Abir Belakehal, Hadjer Djaafer, AbdElkarim Laatamna

**Affiliations:** 1 Faculty of Nature and Life Sciences, Ziane Achour University, P.O. Box 3117, Djelfa 17000, Algeria; bouragba.messaoud@univ-djelfa.dz (M.B.); s.abdellaoui@ens-lagh.dz (S.A.); nesrinegoumich@gmail.com (N.G.); rabiasundes99hbb@gmail.com (S.R.K.); abir.belakehal@univ-tiaret.dz (A.B.); hadjer.djafer2001@gmail.com (H.D.); a.laatamna@univ-djelfa.dz (A.L.); 2 Analytical Biochemistry and Biotechnology Laboratory, Faculty of Biological and Agronomic Sciences, Mouloud Mammeri University, Tizi-Ouzou 15000, Algeria; sarittas694@gmail.com; 3 Biology Department, College of Science, Imam Mohammad Ibn Saud Islamic University (IMSIU), Riyadh 11623, Saudi Arabia; naabdalneim@imamu.edu.sa (N.A.I.); msaleissa@imamu.edu.sa (M.S.A.); 4 Pediatrics Service, Hospital of Ain-Oussara, Djelfa 17000, Algeria; meriemaissou7@gmail.com

**Keywords:** cystic echinococcosis, human hydatidosis, surgical incidence, epidemiology, Djelfa, one health, neglected tropical diseases

## Abstract

Cystic echinococcosis (CE), a neglected zoonotic disease caused by *Echinococcus granulosus* sensu lato, poses a persistent public health burden in agro-pastoral regions worldwide. This study provides a large-scale epidemiological assessment of CE, highlighting sustained zoonotic transmission driven by agro-pastoral practices and human–animal interactions, and supporting the urgent implementation of One Health strategies. This ten-year retrospective study (2015–2024) analyzed 326 surgically confirmed cases from five hospitals in Djelfa. The cumulative surgical incidence was 2.04 cases per 100,000 person-years, classifying the region as hypoendemic. Females predominated (61.96%), and individuals aged 31–60 years represented 47.24% of cases. Rural residence (73.62%) and dog contact (94.17%) were major risk factors, with hepatic localization dominating (88.96%). Correlation analysis showed moderate associations between rural habitat and dog contact (V = 0.46, *p* < 0.001) and between sex and habitat (V = 0.34, *p* < 0.001), as well as weaker but significant associations for age and cyst location (V = 0.28, *p* < 0.001) and dog contact and cyst location (V = 0.20, *p* < 0.05). No postoperative mortality was recorded. These findings confirm active transmission linked to agro-pastoral practices and emphasize the need for coordinated One Health control strategies.

## 1. Introduction

Cystic echinococcosis (CE), also known as hydatidosis, is a zoonotic parasitic disease caused by the larval stage (metacestode) of *Echinococcus granulosus* sensu lato (s.l.) [1]. It represents a major public health and economic concern worldwide [2,3] and is currently classified among the 20 neglected tropical diseases (NTDs) prioritized by the World Health Organization (WHO) [4]. Globally, CE affects more than one million people and is responsible for approximately 19,300 deaths each year, with an estimated annual economic loss exceeding USD 3 billion, accounting for both direct human healthcare costs and livestock-related losses [5,6]. The annual incidence typically ranges from 5 to 10 cases per 100,000 inhabitants, although rates as low as 1 and as high as 25 per 100,000 have been reported in low-endemic and highly endemic areas, respectively [1,2,4]. The cost of surgical intervention per patient may range from USD 1000 to 10,000 depending on disease severity and complications, imposing a substantial burden on health systems in resource-limited settings [7,8,9,10,11].

The life cycle of *E. granulosus s.l.* involves two mammalian hosts. Adult tapeworms reside in the small intestines of definitive hosts, primarily domestic dogs and other canids, while larval stages develop as hydatid cysts in the internal organs of intermediate hosts, mainly herbivorous and omnivorous animals such as sheep, cattle, and camels [7,8]. Humans act as accidental intermediate hosts by ingesting parasite eggs shed in the feces of infected dogs, typically through contaminated soil, water, vegetation, or direct contact with infected animals [12]. Diagnosis of human CE relies on a combination of imaging techniques with ultrasound as the first-line tool, complemented by computed tomography and serological testing using immunodiagnostic assays such as ELISA for the detection of specific anti-Echinococcus antibodies [13,14].

While CE poses a global health challenge, its burden is disproportionately high in regions where the parasite’s life cycle is sustained by traditional livestock farming practices. This is particularly true across North Africa, including Algeria, where the disease constitutes a persistent veterinary and public health challenge. Slaughterhouse-based surveys have revealed high infection rates in livestock throughout the country, with prevalence reaching up to 78% in sheep, 91% in cattle, and 26% in dromedary camels in certain provinces [15,16]. Molecular investigations have confirmed the predominance of *E. granulosus* sensu stricto (s.s.) in both livestock and human cases across the country, while the camel strain (G6) has also been identified in dromedaries and, more rarely, in human infections from Saharan regions [17,18,19,20,21]. In contrast, epidemiological data in humans remain scarce and fragmented, yielding an estimated surgical incidence ranging between 1.5 and 2.5 cases per 100,000 inhabitants [8,20,21].

Despite this context, comprehensive epidemiological data on human CE in Algeria remain scarce, geographically fragmented, and largely concentrated in northern urban centres. The steppe and semi-arid regions of the country where agro-pastoral activities are central to the local economy and where conditions for parasite transmission are particularly favorable remain poorly documented. Consequently, the true burden of the disease in high-risk areas remains unknown, hindering the development of targeted control strategies. The province of Djelfa, situated in the heart of the Algerian steppe and home to one of the largest pastoral populations in the country, is emblematic of this gap. With livestock farming employing nearly 36% of the local workforce and extensive contact between rural communities, dogs, and sheep, Djelfa presents an epidemiological profile conducive to sustained CE transmission. To date, only one limited study from this province has been published [19], providing data on eleven surgically confirmed cases over a single year.

To address this gap, the present study aimed to conduct a comprehensive ten-year retrospective analysis (2015–2024) of human CE cases diagnosed and surgically treated across five public hospitals in the Djelfa region. Specifically, it sought to: (i) estimate the surgical incidence of CE; (ii) describe the epidemiological and clinical profile of affected patients; (iii) identify key risk factors associated with infection; and (iv) explore, through a correlation matrix approach, the relationships between these different risk factors in order to identify statistically significant associations among epidemiological determinants. By achieving these objectives, this research aims to provide updated and methodologically robust regional data to inform future surveillance strategies and evidence-based control efforts in this endemic agro-pastoral setting.

## 2. Material and Methods

### 2.1. Ethical Approval Statement

Ethical approval for this retrospective study was obtained from the Ethics Committee of the participating hospitals in Djelfa Province (Approval No.: 1405N2/2015).

### 2.2. Study Area

**Figure 1:**
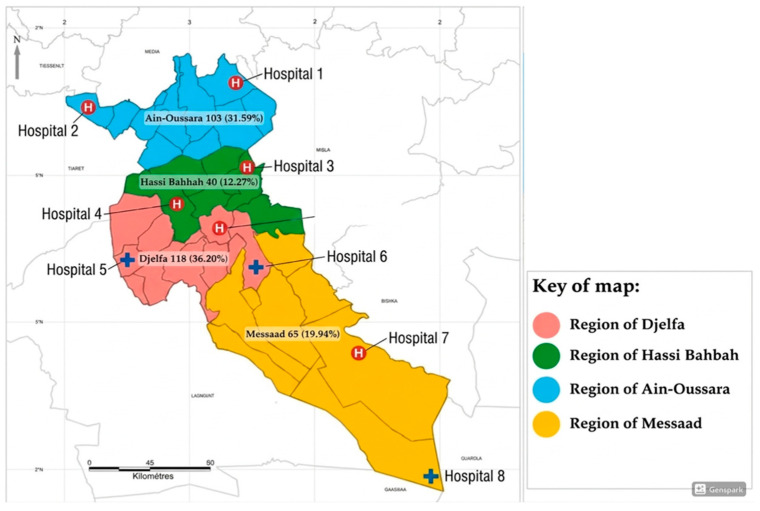
Geographical distribution of CE surgery cases in Djelfa. *Administrative boundaries approximated from OpenStreetMap data (© OpenStreetMap contributors, ODbL)*.

This study was carried out in five public hospitals located in the province of Djelfa. The geographical distribution of surgically confirmed CE cases across the province is illustrated in Figure 1. The province spans an area of 32,256.35 km^2^, accounting for approximately 1.36% of the country’s total surface area. Djelfa is administratively divided into 12 districts (dairas) and 36 municipalities, with a total population exceeding 1.6 million inhabitants. The population is primarily concentrated in the provincial capital, Djelfa city, as well as in the districts of Ain Oussara, Hassi Bahbah, and Messaad (Figure 1).

Djelfa province is served by a total of eight hospitals, comprising five public and three private institutions, strategically distributed across its main administrative districts (Dairas). The capital city of Djelfa houses two public hospitals and three private hospitals, all offering various types of surgical care. The districts of Ain Oussara, Hassi Bahbah, and Messaad each contain one public hospital, ensuring regional access to essential healthcare services [15].

The population of Djelfa is estimated at 1,626,210 inhabitants, with an average annual growth rate of 4.08%. The average population density stands at 50.51 inhabitants per square kilometer. Males slightly outnumber females, with 836,140 and 790,070 individuals, respectively. The province has a predominantly young population: 36.86% (599,365 individuals) are under the age of 15, and 32.35% (526,120) fall within the 15–29-year age group. The working-age population (30–59 years) accounts for 25.35% (412,461), while those aged 60 years and above represent 5.44% (88,264) of the total [15,19].

In terms of spatial distribution, 81.4% of the population (1,324,177 individuals) resides in primary and secondary urban areas, while 18.6% (302,033 individuals) live in rural zones [19].

From an economic standpoint, Djelfa benefits from extensive natural resources, particularly its vast steppe pastures, which cover more than 2 million hectares approximately 66.24% of the province’s total surface area. This ecological feature supports a predominantly agro-pastoral economy. Agriculture and livestock rearing employ nearly 36% of the workforce, followed by employment in public administration (29%), construction and public works (17%), commerce (14%), and industry (4%) [15,19].

### 2.3. Data Collection

This study is based on a ten-year retrospective analysis (2015–2024) aimed at compiling and analyzing epidemiological data from patients diagnosed with cystic echinococcosis (CE) and surgically treated in five public hospitals within the Djelfa region. The diagnosis of CE was established through a combination of imaging techniques, primarily ultrasound, and serological confirmation using a commercial anti-*Echinococcus granulosus* IgG ELISA kit (NovaLisa^®^ *Echinococcus* IgG ELISA, product code ECHG0130, NovaTec Immundiagnostica GmbH, Dietzenbach, Germany), with definitive confirmation obtained intraoperatively during surgery.

Patient data were extracted from hospital medical records and included demographic and clinical variables such as age, sex, place of residence (urban or rural), anatomical localization of the hydatid cysts, history of contact with dogs, and clinical outcome (recovery or death) following surgical intervention.

### 2.4. Statistical Analysis

Epidemiological data from cystic echinococcosis (CE) cases were analyzed using STATISTICA software (version 6.1). Categorical variables were described as frequencies and percentages. The distribution of CE cases according to demographic and epidemiological characteristics (sex, age, habitat, cyst localization, and dog contact) was first assessed using the Chi-square (χ^2^) goodness-of-fit test. Subsequently, associations between these variables were examined using the Chi-square (χ^2^) test of independence, and their strength was evaluated using Cramér’s V coefficient. To provide an overall view of the relationships between variables, a matrix of pairwise associations was generated and visualized using a heatmap. Statistical significance was set at *p* ≤ 0.05, with a 95% confidence level.

## 3. Results

### 3.1. Spatio-Temporal Distribution of Human Cystic Echinococcosis Cases

A total of 326 CE cases were recorded between January 2015 and December 2024 in the five hospitals of Djelfa. The annual average number of patients operated on was 32 in this region, giving an overall surgical incidence of around 2.04 cases per 100,000 person-years. The most CE cases were recorded in the hospital of Djelfa, followed by the hospital of Ain-Oussara. A significant difference was observed between the distribution of CE cases and the hospital where these cases were found (Table 1).

### 3.2. Epidemiological Profile of Operated Hydatid Cyst Patients

Table 2 summarizes the epidemiological profile of the 326 patients who underwent surgery for CE. Among them, 202 (62%) were female and 124 (38.04%) were male, revealing a statistically significant predominance of cases among females (*p* < 0.001), with a sex ratio of 1.63 in their favor.

The mean age of the patients was 39.23 years, ranging from 8 to 78 years. The most affected age groups were 31–60 years (47.24%) and 17–30 years (30.98%), with the distribution across age categories showing a significant difference (*p* < 0.001).

Regarding geographical origin, the majority of patients (240 out of 326; 73.62%) lived in rural areas, a statistically significant result (*p* < 0.001) highlighting the strong link between CE and rural environments. Moreover, a vast majority of patients (94,17%) reported close contact with dogs during childhood, while only 5,83% had no such exposure, confirming a significant association between canine contact and CE transmission (*p* < 0.001).

In terms of cyst localization, the liver was by far the most frequently affected organ, accounting for 290 cases (88.96%), followed by the lungs (25 cases; 7.67%) and other organs such as the kidneys, uterus, and spleen (11 cases; 3.37%). These distributions were also statistically significant (*p* < 0.001).

Notably, no postoperative deaths were reported throughout the entire study period, and all patients recovered successfully following surgery.

### 3.3. Association Between Epidemiological and Clinical Variables

**Figure 2:**
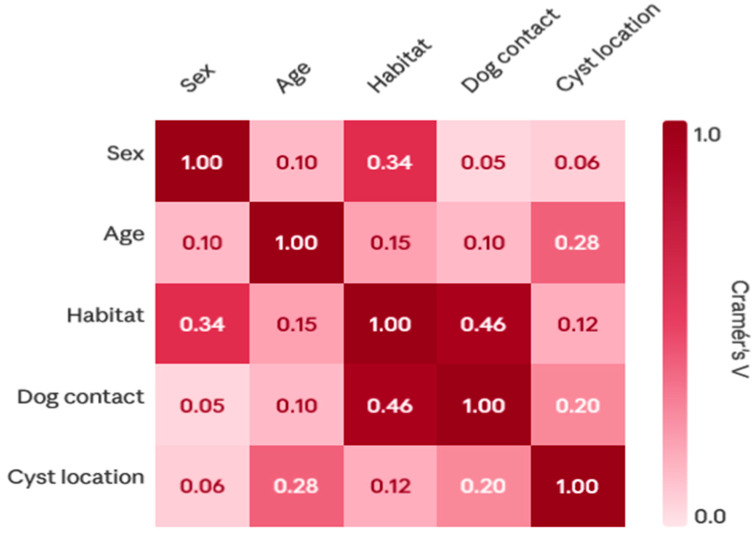
Heatmap of Cramér’s V coefficients for associations between risk factors (sex, age group, habitat, dog contact, and cyst location).

The strength of associations between categorical risk factors was assessed using Cramér’s V (Table 3). The strongest associations were observed between rural habitat and dog contact (V = 0.46, *p* < 0.001), followed by sex and habitat (V = 0.34, *p* < 0.001), and age with cyst location (V = 0.28, *p* < 0.001). A weak association was found between dog contact and cyst location (V = 0.20, *p* < 0.05). All other associations were very weak and not statistically significant. A heatmap of Cramér’s V coefficients was generated to visualize these associations (Figure 2), providing a clear overview of the interrelationships among key epidemiological and clinical risk factors.

## 4. Discussion

Human cystic echinococcosis (CE) remains a major yet neglected zoonotic disease, posing significant public health challenges. Its epidemiology is still insufficiently characterized, mainly due to the lack of comprehensive data and the absence of a national surveillance system [19]. The present retrospective study provides updated epidemiological insights into human CE in an endemic region. To date, only a limited number of epidemiological investigations have been conducted in Algeria. Table 4 summarizes the available literature on CE across the country between 1926 and 2024 [17,18,20,21,22,23,24,25,26,27,28,29,30,31,32,33,34,35,36,37,38,39,40,41].

As highlighted in Table 4, epidemiological data on human CE in Algeria remain geographically fragmented and are largely concentrated in northern urban centers, with very few studies addressing steppe and semi-arid regions. In this context, our study fills an important gap by providing the first comprehensive ten-year dataset from the Djelfa region. The overall surgical incidence was estimated at 2.04 cases per 100,000 person-years, which is consistent with the national range of 1.5 to 2.5 cases per 100,000 inhabitants reported in Algeria [2,17,18,20,21]. According to internationally accepted endemicity thresholds, this incidence classifies Djelfa as a hypoendemic area (incidence < 7.5 cases per 100,000 person-years), placing it in a comparable epidemiological context to other agro-pastoral regions of the Mediterranean basin, such as Libya, Morocco, and Turkey [42,43,44,45]. The hypoendemic status observed in the study area, despite the likely high level of infection in livestock, may be explained by the predominance of underdiagnosis and the asymptomatic nature of cystic echinococcosis, leading to underreporting of cases [42]. In addition, differences in slaughtering practices, partial interruption of the parasite life cycle, and variable dog–livestock–human contact intensity may contribute to limiting human transmission [44,45]. It is also possible that infection in livestock does not always translate into efficient human exposure, depending on local hygiene practices and dog management systems.

However, this relatively low incidence likely underestimates the true burden of the disease. Indeed, the exclusive reliance on surgically treated cases excludes asymptomatic individuals, patients managed conservatively (PAIR or pharmacological treatment), and those who did not seek medical care. In addition, limited access to specialized healthcare in rural areas and the potential for undiagnosed cases may further contribute to underreporting. These findings highlight the need to strengthen epidemiological surveillance systems and improve diagnostic capacities to obtain a more accurate estimation of CE burden in the region.

A significantly higher proportion of CE cases was observed in rural areas compared to urban settings, with 73.62% of patients originating from rural communities (*p* < 0.001). This finding is consistent with studies conducted in Algeria, Libya, and Morocco, which report a higher prevalence of CE in rural environments [17,42,43]. This pattern can be explained by ecological and behavioral factors that favor parasite transmission, particularly the close interactions between humans, dogs (definitive hosts), and livestock (intermediate hosts), especially sheep. The moderate association observed between rural habitat and dog contact (V = 0.46) further supports the role of the agro-pastoral lifestyle in maintaining the transmission cycle of *Echinococcus granulosus* [25,42,43].

In the specific context of Djelfa, the vast steppe landscape and the predominance of transhumant pastoralism create ecological conditions highly favorable to parasite circulation. Additionally, practices such as unsupervised home slaughter, improper disposal of infected offal, and limited awareness of preventive measures contribute to sustained transmission in rural settings [19]. Nevertheless, CE transmission in urban areas should not be overlooked. In peri-urban and underdeveloped neighborhoods, poor sanitation and the presence of stray dogs may also facilitate disease spread. Furthermore, increasing human and animal mobility between rural and urban areas may contribute to the emergence of CE in cities, a trend increasingly reported in endemic countries.

Consistent with our findings, several studies have reported a higher proportion of CE cases among females. In our cohort, 61.96% of patients were female. A moderate association between sex and habitat (V = 0.34) indicates that women were more frequently represented in rural areas. This distribution may partly explain the higher proportion of female cases observed, particularly given the strong association between rural residence and dog contact. In contrast, the direct association between sex and dog contact was weak and not statistically significant (V = 0.05, *p* > 0.05), suggesting that the higher frequency of CE in women is not related to sex-specific exposure, but rather to their greater representation in higher-risk rural environments. The predominance of female cases may be explained by socio-cultural and occupational patterns in the study region. In rural and agro-pastoral settings, women are more frequently involved in domestic activities such as food preparation, handling raw vegetables, water collection, and close contact with domestic dogs, which may facilitate accidental ingestion of *Echinococcus granulosus* eggs [19]. In addition, women may have prolonged exposure to contaminated household environments where infected dogs are present, increasing the risk of infection over time [25].

However, other studies have reported a higher prevalence in males [18,46,47], indicating that gender-related risk may vary depending on socio-cultural and occupational contexts. For instance, in peri-urban or industrial settings, men may be more exposed through activities such as shepherding or slaughterhouse work, where handling and evisceration of livestock can lead to direct contact with infected offal, increasing the risk of exposure to *Echinococcus granulosus* [46]. Slaughterhouse workers are particularly at risk due to direct contact with infected offal during livestock evisceration, while inadequate hygiene practices may further facilitate environmental contamination and indirect transmission, especially in settings where dogs have access to discarded infected viscera [46,47]. These variations highlight the importance of context-specific risk assessments and gender-sensitive prevention strategies.

Dogs play a central role in the transmission of *E. granulosus*, acting as definitive hosts that shed parasite eggs through their feces. Our results showed a significant association between CE and frequent contact with dogs, particularly in the absence of regular deworming practices (94.17%, *p* < 0.001), in agreement with previous studies [25,48]. In Djelfa, dogs are commonly used for herd management, resulting in frequent and prolonged contact with humans. The weak association observed between dog contact and cyst location (V = 0.20) suggests that while exposure to dogs is a key factor for infection, it does not influence the anatomical distribution of cysts.

These findings emphasize the urgent need for integrated control strategies. From a One Health perspective, effective CE control requires a coordinated, multi-sectoral approach involving regular dog deworming, management of stray dog populations, regulation of slaughtering practices, veterinary surveillance, and community education. Such approaches have proven successful in countries such as Cyprus, China, and New Zealand [4].

The mean age of patients in this study was 39.23 years, with individuals aged 31–60 years being the most affected group (47.24%). This distribution is consistent with reports from Algeria, Morocco, and Iran [17,43,49], and reflects the well-established pattern of CE predominantly affecting adults of working age in endemic areas. In contrast, studies conducted in Spain and France reported higher mean ages, likely reflecting imported cases among older populations rather than active local transmission [46,50]. The relatively low proportion of pediatric cases observed may be explained by the slow growth of hydatid cysts and the long asymptomatic period of the disease, resulting in delayed diagnosis [20,22]. This latency suggests that preventive interventions targeting younger populations may only yield measurable epidemiological benefits over the long term.

In our study, the liver was the most frequently affected organ (88.96%), followed by the lungs (7.67%) and other organs such as the kidneys, uterus, and spleen (3.37%). These findings are consistent with most reports in the literature [19,21,25,42,43,44,46,47,50]. Instead of making a strict age-related biological assumption, the predominance of hepatic involvement may be explained by the natural filtration role of the liver in parasite dissemination; however, the distribution of cysts in younger patients may also be influenced by factors such as host immunity, parasite load, and the time required for cyst development, which can vary between individuals. Therefore, age-related differences should be interpreted cautiously. A moderate association between age and cyst location (V = 0.28) was observed, reflecting the predominance of hepatic cysts in adults and pulmonary cysts in younger patients. This pattern is well documented and is attributed to the greater elasticity of lung tissue in children, which facilitates cyst development [42,51,52].

In contrast, studies by Zait et al. [18,22] reported a predominance of pulmonary localization. This discrepancy is likely due to referral bias related to the pulmonology specialization of the hospital where those studies were conducted, rather than a true epidemiological difference. Although less common, atypical localizations (kidneys, uterus, spleen) were also observed and accounted for 3.37% of cases. While generally reported at low frequencies, these forms are clinically important due to their diagnostic complexity and increased surgical risk. The absence of a significant association between habitat and cyst location (V = 0.12, *p* > 0.05) further suggests that, once infection occurs, cyst distribution is more influenced by host-related factors than environmental conditions [18,22].

This study highlights the need for integrated control strategies for cystic echinococcosis in agro-pastoral settings such as Djelfa. The strong association between rural residence, dog contact, and disease occurrence underscores the importance of a One Health approach targeting the human–animal–environment interface. Priority measures include regular deworming of dogs, improved slaughterhouse practices, proper disposal of infected offal, and community education on transmission risks. Strengthening surveillance and enhancing collaboration between veterinary and public health sectors are essential for sustainable control. Successful programs in endemic countries demonstrate that such integrated strategies can significantly reduce transmission.

Finally, no postoperative deaths were recorded among the 326 patients included in this study, and all cases had favorable surgical outcomes. Although encouraging, this finding should be interpreted cautiously due to the lack of long-term follow-up data, which are essential for assessing recurrence rates and postoperative complications. Several limitations should be acknowledged. As a hospital-based retrospective study limited to surgically treated cases, the dataset is subject to selection bias and likely underestimates the true prevalence of CE. In addition, the absence of molecular data (molecular typing) limits the ability to characterize transmission dynamics and identify circulating strains. Furthermore, the lack of follow-up data (recurrence) prevents assessment of postoperative recurrence and long-term outcomes. Despite these limitations, this study represents the most comprehensive epidemiological assessment of human CE in the Djelfa region to date and provides a valuable baseline for future research and control strategies.

## 5. Conclusions

This ten-year retrospective study provides the most comprehensive epidemiological assessment of human cystic echinococcosis conducted to date in the Djelfa region, documenting 326 surgically confirmed cases and a cumulative surgical incidence of 2.04 cases per 100,000 person-years. The epidemiological profile characterized by female predominance, concentration among working-age adults, overwhelming hepatic localization, and strong links between rural residence and dog contact collectively reflects an active transmission cycle rooted in the agro-pastoral character of the region. Correlation analysis further demonstrated that rural habitat and dog contact are the most interconnected risk factors, reinforcing the ecological and behavioral drivers of CE transmission in this steppe environment. Taken together, these findings confirm Djelfa as a CE-endemic area and expose critical gaps in surveillance, diagnosis, and disease control. Addressing these gaps urgently requires an integrated One Health response encompassing systematic dog deworming, regulation of home slaughter practices, stray dog population management, and targeted community health education. Future research should prioritize population-based screening, molecular strain characterization, and long-term patient follow-up to better quantify the true disease burden and design evidence-based control programs fully aligned with WHO recommendations for neglected tropical diseases.

## Figures and Tables

**Table 1:** Distribution of CE cases by year and by hospital in Djelfa.

Years	CE Cases by Region and by Year				
	Djelfa	Hassi Bahbah	Ain-Oussara	Messad	Total
2015	9	6	12	6	33
2016	8	7	10	12	37
2017	10	5	8	5	28
2018	9	3	15	5	32
2019	13	6	11	3	33
2020	13	3	11	9	36
2021	14	2	9	6	31
2022	16	3	8	6	33
2023	14	2	11	8	35
2024	12	3	8	5	28
---	118	40	103	65	326

**Table 2:** Epidemiological profile of CE surgery cases (n = 326) in Djelfa between 2015 and 2024.

Variable	Category	Sample Size (n)	Prevalence (%)	X^2^	*p*-Value
Gender	Male	124	38.04	313.34	<0.001
	Female	202	61.96		
Age category	1–16 years	25	7.67	163.37	<0.001
	17–30 years	101	30.98		
	31–60 years	154	47.24		
	61–78 years	46	14.11		
Habitat	Rural	240	73.62	275.44	<0.001
	Urban	86	26.38		
Localization of cysts	Liver	290	88.96	506.46	<0.001
	Lungs	25	7.67		
	Others	11	3.37		
Contact with dogs	Yes	307	94.17	217.76	<0.001
	No	19	5.83		

**Table 3:** Associations between risk factors in patients operated for cystic echinococcosis (n = 326).

Variable Pair	χ^2^	*p*-Value	Cramér’s V
Sex × Age	3.26	>0.05	0.10
Sex × Habitat	42.15	<0.001	0.34
Sex × Dog contact	0.89	>0.05	0.05
Sex × Cyst location	1.17	>0.05	0.06
Age × Habitat	7.33	>0.05	0.15
Age × Dog contact	3.26	>0.05	0.10
Age × Cyst location	35.62	<0.001	0.28
Habitat × Dog contact	68.42	<0.001	0.46
Habitat × Cyst location	5.12	>0.05	0.12
Dog contact × Cyst location	6.84	<0.05	0.20

**Table 4:** Human CE cases reported in Algeria between 1926 and 2024.

Author Name (Reference)	Year of Study	Region (Province)	N. of Samples
Sevenet and Witas [27]	1900–1925	Northern Algeria	1844
Mokhtari [28]	1963–1964	Northern Algeria	866
Larbaoui and Alloula [29]	1966–1970	Northern Algeria	2083
Seimenis [20]	1997–2000	Northern Algeria	648
Gasmi [30]	2005	Setif	1
Tliba et al. [31]	2008	Annaba	3
Fendri et al. [32]	2008–2009	Constantine	7
Yacoubi B.et al. [33]	2012	Algiers	2
Zait et al. [17]	2006–2011	Algiers	209
Zait et al. [18]	2005–2012	Algiers	78
Hamouda et al. [34]	2015	Batna	1
Samai and Bouaziz [35]	2009–2014	Annaba	7
Zait et al. [22]	2012–2014	Algiers	54
Lakehal et al. [36]	2000–2016	Constantine	25
Baya et al. [37]	2019	Constantine	1
Boukerroucha et al. [38]	2000–2018	Annaba	27
Boukerroucha et al. [39]	2021	Annaba	1
Moussa et al. [21]	2021	Western Algeria	46
Laatamna et al. [19]	2020	Djelfa	11
Bouchenaki et al. [40]	2023	Algiers	1
Messaoudi [41]	2024	Ouargla	1

## Data Availability

The data sets during and/or analyzed during the current study are available from the corresponding author upon reasonable request.
